# What do people agree to when stating willingness to donate? On the medical interventions enabling organ donation after death

**DOI:** 10.1371/journal.pone.0202544

**Published:** 2018-08-24

**Authors:** Linda Gyllström Krekula, Ulla Forinder, Annika Tibell

**Affiliations:** 1 Function area—Social Work in Health Care, Karolinska University Hospital, Stockholm, Sweden, Department of Learning, Informatics, Management and Ethics (LIME), Karolinska Institutet, Stockholm, Sweden; 2 Department of Neurobiology, Care Sciences and Society (NVS), Karolinska Institutet, Stockholm, Sweden; University of Gävle, Department of Social Work and Psychology, Gävle, Sweden; 3 Program Management Office (PMO), New Karolinska, Karolinska University Hospital, Stockholm, Sweden, Department of Learning, Informatics, Management and Ethics (LIME), Karolinska Institutet, Stockholm, Sweden; University of Bedfordshire, UNITED KINGDOM

## Abstract

**Purpose of the study:**

The purpose of this study is to explore donor relatives’ experiences of the medical interventions enabling organ donation, as well as to examine the donor relatives’ attitudes towards donating their own organs, and whether or not their experiences have influenced their own inclination to donate.

**Methods:**

The experiences of donor relatives were explored via in-depth interviews. The interviews covered every step from the deceased family member being struck by a severe bleeding in the brain till after the organ recovery, including the medical interventions enabling organ donation. The interviews were analysed through qualitative and quantitative content analysis.

**Results:**

Brain death and organ donation proved to be hard to understand for many donor relatives. The prolonged interventions provided after death in order to enable organ donation misled some relatives to believe that their family member still was alive. In general, the understanding for what treatment aimed at saving the family member and what interventions aimed at maintaining organ viability was low. However, most donor relatives were either inspired to, or reinforced in their willingness to, donate their own organs after having experienced the loss of a family member who donated organs.

**Conclusions:**

There is a need for greater transparency regarding the whole chain of events during the donation process. Yet, having experienced the donation process closely did not discourage the donor relatives from donating their own organs–but rather inspired a willingness to donate. This indicates an acceptance of the medical procedures necessary in order to enable organ donation after death.

## Introduction

The purpose of the medical care at an intensive care unit (ICU) is mainly to ease pain, to cure and to save lives. Nevertheless, some patients cannot be saved and death is inevitable. A limited number of patients then have the possibility to contribute to saving the lives of other patients by donating their own organs after death.

In this article, we describe the experiences of donor relatives with regard to medical treatment transitioning from being provided in order to save the life of a patient to being provided in order to make organ donation possible. Taking part of the donor relatives’ unique experiences of the circumstances surrounding organ donation offers us the opportunity to evaluate and improve the management of the delicate donation process.

All care and treatment provided within our medical health care should be provided with the informed consent of the patients and with the best interests of the individual patient in mind [1]. This raises the question of how the measures taken during the donation process relate to the patient’s best interests. In some countries, e.g. the U.K. [2], in the case of a patient lacking the capacity to make decisions, the recommendation is “*… to establish whether taking steps*, *before death*, *to facilitate organ donation would be in the patient’s best interests*” [3]. Hence, an attempt to establish the patient’s decision regarding organ donation is made, and the medical interventions provided in order to enable organ donation are then regarded as being in the patient’s best interests. In countries where a request for a patient’s intentions regarding organ donation cannot be made till after the death of the patient, the prolongation of care becomes more complex, as it cannot be established whether the organ-preserving interventions before death really are in the patient’s best interests. In some countries, like for example Sweden, national provisions state that all life-sustaining treatment should be withdrawn before death, when the treatment no longer is benefitting the patient and instead is prolonging the dying process, causing pain and suffering [4, 5]. This further adds to the complexity of the situation as the interventions enabling organ donation needs to be prolonged until brain death if an organ donation shall be possible [4–7]. This conflict, between withdrawing or prolonging treatment is currently addressed in a government investigation [8].

Brain death, defined as the brain having lost all brain functions, totally and irreversibly, has been the criteria for death since 1987 in our county Sweden. Though this concept of death is widely accepted, it sometimes complicates the understanding of death. In the situation of organ donation, the patient dies while treated with a ventilator, which means that the dead patient looks alive although being dead. However, continued ventilator treatment is the prerequisite for donation after brain death, to maintain organ viability.

In order to maintain public trust in the donation and transplantation system, clarity and transparency of all the actions taken during the donation process is fundamental [7, 9]. According to the *Eurobarometer* conducted in 2009, which is a survey commissioned by the European Commission's Directorate General SANCO, that included 26,788 European citizens in the 27 European Union Member States, as well as 3,504 in the candidate countries and the Turkish Cypriot Community, the main reasons for people not wanting to donate organs were fear of manipulation of the body and distrust in the system [10]. Hence, the extent of the general public’s trust is reflected in its degree of willingness to donate. Findings regarding distrust were much less frequent in the Northern European countries compared with some of the Eastern European countries [10]. In our country, Sweden, the level of trust was very high, which tallies with a widespread willingness to donate [10]. Furthermore, the willingness to donate is more widespread in our county than in any other European country included in the barometer, with 83 percent of the public stating to be willing to donate [10]. However, what we do not know is what actually is comprised in a stated willingness to donate. Most people probably realise that a donation requires surgical procedure, but do people actually realise that a donation also requires prolonged ICU-care? Are people prepared to go through specific medical procedures, before as well as after being pronounced brain dead, in order to be able to donate organs? Would a deeper understanding of the medical procedures surrounding organ donation have an impact on an individual’s inclination to donate?

Though the trust for the health care, as well as the willingness to donate is high in our country, we are still struggling with relatively low donation rates, and people are still dying while waiting for an organ [10, 11]. However, the donation rates have increased from 14.7 per million inhabitants in year 2012 to 18.5 in year 2016 [11]. Nevertheless, there is a need for a greater understanding of what aspects that may influence the donation rates.

One subgroup of the general public who is well acquainted with organ donation due to their presence during the donation process and who has witnessed the medical interventions enabling organ donation, is the donor relatives. Consequently, this group has a unique insight into the procedures at our ICUs and can therefore provide us with important knowledge of the interventions enabling organ donation.

The aim of this study is to explore donor relatives’ experiences of the medical interventions enabling organ donation, as well as to explore the donor relatives’ attitudes towards donating their own organs, and whether or not their experiences have influenced their own inclination to donate.

## Methods and participants

### Inclusion criteria

**Fig 1:**
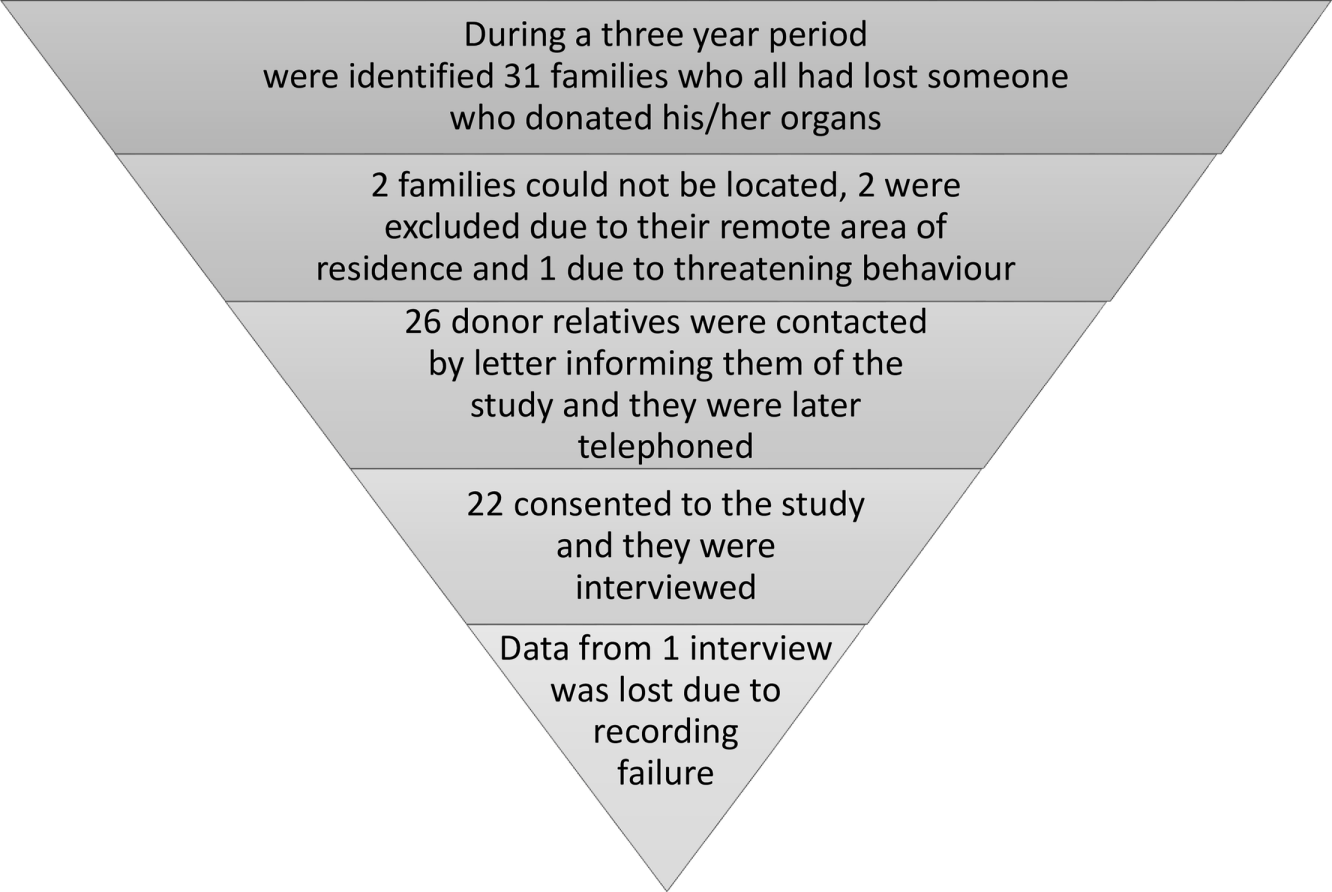
Inclusion of participants (N 21).

**Fig 2:**
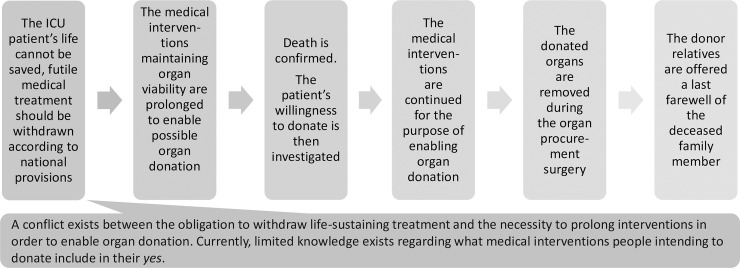
Phases of the donation process included in the analysis.

In this interview study we included relatives of patients who died due to a total brain infarction and who donated their organs. The families in the study were all relatives of patients at the same Neuro Intensive Care Unit at a University Hospital, in Stockholm Sweden, during the years 2001–2004 (Fig 1). The donation process studied, and the regulations has not changed since the study period. This group was chosen to obtain the most informative participants (purposive sampling) [12]. Hence, the included participants were chosen on the basis of their having experienced the whole donation process, including the non-therapeutic intensive treatment preparing for organ donation. The phases of the donation process that are of interest for the study are illustrated in Fig 2.

An ethics application was sent to the regional ethics committee in Stockholm. The ethics committee stated that the research was not covered by the Ethics Review Act. This, as the research was carried out after the participants had given due consent to the treatment. Therefore, no formal decision was made by the ethics committee, but the authors received a recommendation from the committee in which the committee stated that it did not find any obstacles for the study to be carried out (ref number 04-615/5). Nevertheless, when planning and conducting a study like this one there are several ethical considerations that need to be made. The authors’ ethical considerations are described in the appendix.

### Data collection

To guarantee coverage of the same topics with all participants, data were collected using a semi-structured interview guide with open-ended questions (see interview guide). The interviews covered every step from the deceased family member being struck by a life-threatening brain haemorrhage, to the stay at the ICU including the whole donation process, to the hospital follow-up and the nearest future after that. The interviews also included questions about the donor relatives’ own decision-making process regarding donating their own organs. The interviews were conducted by the first author, who is an experienced medical social worker specialised in organ donation and transplantation. The interviewer had had no prior contact with the included donor relatives.

All interviews but three were conducted in the participants’ homes, the location was chosen by the participants. The interviews were carried out in Swedish and they were recorded and transcribed verbatim. All interviews were conducted with the informed consent of the participants. All participants spoke Swedish fluently, 20 of them came from Sweden and one from Finland. None of the participants expressed any strong religious beliefs in the interviews. About one third of the participant had some sort of higher education. For further demographics of the participants and interview information, see Table 1.

**Table 1:** Included relatives, interview information and participant demographics.

Included relatives	Gender	Age	Lost	Cause of death	Time between death and interview	Duration of the interviews
N = 21	Men: 10	Mean: 55	Partner/spouse: 13	Accident: 3	Mean: 3,1 years	Mean: 1h, 7 min
	Women: 11	Median: 55	A child: 4	Stroke: 18	Median: 2,7 years	Median: 1h, 5 min
		Max: 84	A parent: 3		Max: 6,7 years	Max: 2h, 6 min
		Min: 29	A sibling: 1		Min: 1,4 years	Min: 40 min

### Analysis

In order to fulfil the aim of this study, the methodologies used in the analyses are both qualitative and quantitative content analyses [12–15]. Inductive qualitative approach was used in order to explore the underlying meaning of the donor relatives’ experiences of the medical interventions enabling organ donation. When we conducted the analysis and interpreted the donor relatives’ experiences of the medical interventions enabling organ donation, it became clear that we needed to understand what interventions that the donor relatives’ thought was provided to cure or save the patient’s life, and what interventions that they believed was provided to make organ donation possible. This analysis led us to the first main category that we call “from life sustaining to organ preserving interventions”. To gain an understand for this transition, we also needed to understand when the donor relatives believed that their family member had died. This led us to the second main category, “the occurrence of death”. The end point of the medical interventions enabling organ donation, that derive from the interviews, constitutes the third main category, “the donor operation”.

To fulfil the aim of the study, we also needed to understand whether the donor relatives’ decisions on organ donation were influenced by the donation process in conjunction with the loss of a family member who donated organs. This led us to the fourth main category, “the donor relatives’ own decision-making process”. All main- and subcategories that derive from the qualitative content analysis are illustrated in Table 2, exemplified with quotes from the interviews.

**Table 2:** Illustration of the relation between main categories, categories and subcategories.

Main categories	Categories	Subcategories	Exemplifying quote
**The occurrence of death**	Time point for death according to donor relatives	- Immediately when collapsing - During determination of brain death - Formally informed about death - At donation request - During donor operation - When the heart stopped beating	*“*.* *.* *.*I sat there by the ambulance and then I kind of understood*, *oh…is it this bad… Because I saw that it was just*, *that she wasn’t there so to say*. *No*, *I think that she was dead already then*.*”*
		Uncertain / Contradictory: - Staff communication with dying and dead patient - Vague information about death - Psychological difficulties accepting death	“*She didn’t say it explicitly–dead–but that there was no reaction up here in the brain*. *And then one can figure out that it must be something like that*.*”*
	Mistrust regarding declaration of death	- Insufficient skills	*“…what if they didn’t do it correctly*. *One has read*, *sometimes one reads about things…”*
**From life sustaining to organ preserving interventions**	Ventilator treatment after death	- Kept patient alive - Maintained though *no hope* - Maintained even *though dead* - Maintained after death for the sake of the organs - Maintained to provide oxygen to the organs - *Prolonged* to enable organ donation	*“He was alive thanks to the machines*, *so without the machines he would have died and then they cannot pick anything from him*.*”*
	Other medical interventions given and preparations made in order to maintain organ viability	- Plenty of preparations - Fluids	*“…they give fluids and various other things to keep the organs fresh”*.
	Suspicion of less good medical treatment	- Now we have a chance - Did not give 100 percent - A done deal	*“Look*, *here we have a chance*, *we might not be able to save him…”*
**The donor operation**	Information about the operation beforehand	Unspecific information: - Vague information from staff - Received no information - Cannot recall any information - Were informed but remember little - Didn’t want information - Reluctance to discuss- Specific surgeons	*“No they did not tell me anything*, *I mean*, *it is their thing…”*
		Specific information: - Related to the organs - Specific surgeons - Logistic planning	*“Well*, *she told us which organs one can use*, *cornea and*…”
	The surgical procedure	- Positive/Respectful - Neutral/Distanced - Negative/Uneasiness	*“It is just like a*, *ehh*, *surgery*. *So they treat you like in any surgery… well like in any operation*. *With respect as well…”*
	The state of the body after the operation	- Positive - Neutral - Negative	*“… that they were going to use the eyes sounded a bit nasty*. *But when we later took farewell of her one could not notice it at all*. *She looked so nice…”“… that they were going to use the eyes sounded a bit nasty*. *But when we later took farewell of her one could not notice it at all*. *She looked so nice…”*
**The donor relatives’ own decision-making process**	Directly affected by their dead family member’s donation	Positively influenced: - Gained knowledge - Inspired by outcome of the donation - Proud of deceased family members’ donation	*And then they told me that they make a decision on a case-by-case basis*. *So*, *you don’t have to be too old at all*, *but that was what I thought before*.*”*
		Negatively influenced: - Treatment mistakes - Media exposure	*”…It was a young doctor and he fiddled with the drainage so*… *No there were so many mistakes…”*
	Mind made up long time ago	Willing: - Willingness to help others - Right thing to do - Organ shortage - Employment in medical health care	*“Well*, *I want to help if I can…”*
		Unwilling: - Fear not dead - Buried according to old traditions	*“I’m too old you know*, *and I come from the country side… I just want to be buried whole according to old traditions*.*”*

In order to count the donor relatives’ own decisions regarding organ donation, a quantitative content analysis was made [12, 16] with three pre-set categories (willing, unwilling, indecisive). We then identified the donor relatives’ retrospective statements regarding their decisions before losing their family member and their decisions after having lost their family member. This allowed us to identify and quantify positive or negative changes in the donor relatives’ willingness to donate after having been involved in the donation process. By combining quantitative and qualitative results the study provides *“more insightful and meaningful results than would using either approach alone”* [15].

To help organize the extensive material, the areas of interest were extracted with the help of the qualitative software programme ATLAS.ti [17]. The use of such programmes enhances the possibility to continuously move from parts of the interviews, to the whole, to the context in a specific interview, which is necessary in order to explore and interpret the underlying meaning of the donor relatives’ statements. When exploring the donor relatives’ statements, this process of constantly moving from parts to the whole and vice versa was continuously applied, in order to validate the interpretations made in the analysis. The qualitative content analysis can be described as a process: from repeatedly reading the entire interviews in order to gain a sense of the whole, to gradually focusing on the content areas that are most essential for the purpose of the study, to dividing these areas into smaller meaning units and coding them with explanatory codes that derive from the text, and sorting the codes into categories and subcategories with internal homogeneity and external heterogeneity. In this paper, the headings correspond with the main categories and the subheadings, or bold text, constitute the subcategories. The results are exemplified with quotes from the interviews to illustrate their origin.

The final analysis was made by the first author/the interviewer. However, one of the co-authors—an experienced qualitative researcher, Professor and Social Worker—also read all the interviews and made an independent analysis. A comparison of the co-author’s and the interviewer’s analysis was then made. In cases of differing analyses, a discussion of how to interpret the findings, codes and categories, took place, until a shared understanding was reached and both authors felt satisfied with the interpretation deriving from the discussion. To test the credibility and the confirmability a continuous dialogue took place within the research team, and between the interviewer and the co-author during the whole process of analysis [12, 13, 18].

## Results

### The occurrence of death

The point of time at which the relatives perceived their family member to have died varied widely: from the time when the family member initially collapsed to the actual donor operation.

Some believed that death had occurred **immediately** when the family member first collapsed, for example at home or at work or in the ambulance on the way to the hospital: “…*I sat there by the ambulance and then I kind of understood*, *oh…is it this bad… Because I saw that it was just*, *that she wasn’t there so to say*. *No*, *I think that she was dead already then*.*”*

Others considered death to have occurred during the process of **brain death being determined** or when they were **formally informed of the declaration of death**: *“…And then the second time*, *well they said that now we have done the next test and there was still no reaction*. *And then they declared him dead and also set a time…”*

For other donor relatives the insight came **when they were asked about organ donation**: “*But then I remember that this nurse came (donation nurse)*, *and then*, *then I understood…”*

Some even believed that the family member died **in connection with the donor operation**: *“…I didn’t see her after the operation*, *or wait… I did see her when she was dead…”*, *“Well yes*, *we knew that it would be over then (after the operation)*, *that he would be dead then…”*. These relatives often believed that death occurred **when the heart stopped beating**: *“That someone is dead*, *that is when the heart stops working… So that…that is an easy thing to know… “*.

Finally, we found relatives who were **uncertain of the time of death** or who talked about it in a **contradictory** manner. The **staff’s communication with the unconscious or, in some cases, the dead patient**, most likely contributed to the uncertainty conveyed by some relatives regarding the patient’s condition: *“…The staff were speaking to him all the time…it was kind of amazing*, *the respect towards a man who really almost could be seen as dead*, *but well*, *you never know…”*,*”Well*, *they were speaking to him…until the very end…”*. Some also stated that they received **vague information about the declaration of death** from the staff: “*She didn’t say it explicitly–dead–but that there was no reaction up here in the brain*. *And then one can figure out that it must be something like that*.*”* There was only one relative who **did not completely trust the reliability of the procedures to declare death**: *“…what if they didn’t do it correctly*. *One has read*, *sometimes one reads about things…”*. We also found relatives who in this demanding situation had **psychological difficulties accepting the loss** and this made it hard to understand that death had occurred: *“She isn’t dead at all*, *and I became somewhat aggressive to him (the physician)… Because the last thing I saw of her*, *was that she still was alive…ehhh…she was alive when I left the emergency room and now all of a sudden she was dead*, *no*, *I couldn’t get it into my head*. *She couldn’t be brain dead—she is alive*. *That’s just the way it is*.*”*

### From life sustaining to organ preserving interventions

#### Being on a ventilator

The only aspect commented on by almost all relatives regarding the medical interventions necessary for organ donation, was the ventilator treatment. However, the understanding of the purpose of the ventilator varied widely.

Some relatives indicated that they thought **the ventilator was keeping their family member alive**—even after the diagnosis of brain death. These relatives were often uncertain about when death had occurred. Some even thought that their family member still was alive at the time of the actual organ recovery: “*He was alive thanks to the machines*, *so without the machines he would have died and then they cannot pick anything from him*.*”* Even though the ventilator made it hard to fully grasp the concept of death, most relatives understood that there was **no hope for recovery despite the use of the ventilator,** because: “*the brain cells were dying”*, *“nothing worked in the brain”*, *“there was nothing to do about it”*,*” there was no return” and “the end was near”*.

Other relatives expressed that their family member **indeed was dead despite the ventilator** and they mentioned that the ventilator was kept on anyway: “*to keep the body going”*, *“to keep the body functions going”*, *“to keep everything possible going”*. However, they were not specific about this being done for the sake of the organs. Another group of relatives were well aware of the necessity to keep their family member on the ventilator with the sole purpose of enabling organ donation in the sense that a **high quality of the organs needed to be maintained**: “*to keep a high quality of the lungs”*,*”to be able to use the heart and all that”*,*”so that the organs would not be destroyed” and”the organs needed to be fresh”*. Some also knew that one of the main factors for the quality of the organs was their **access to oxygen**: “*they needed to keep him breathing to not damage the organs”*, *“it was the organs that needed oxygen so that they could be used”*.

Some also mentioned the necessity to actually **prolong the ventilator treatment** to make an organ donation possible, to gain time to ask about organ donation, to prepare for organ donation, and to conduct the donor operation:”*I do not know for how long–but enough to be able to donate”*,*”it was just for the sake of the organ donation—otherwise they would have switched it off much earlier”*. Generally, the prolonged treatment was not commented on as something that caused discomfort—only two relatives indicated some discomfort: *“She was still connected to all the tubes and she was full with cables everywhere*, *and swollen*, *unpleasant in that way*, *very unpleasant*.*”*, *“It seems like they just awaited death*, *one almost wondered why she had to go there*.*”*

#### Other medical interventions given and preparations made in order to maintain organ viability

The donor relatives hardly talked about any other medical interventions given in order to enable organ donation than the ventilator care. The only statements we found which reflect any other type of interventions were: “*before one ends up on the operating table—there are a lot of* ***preparations*** *that need to be done”*, *“they give* ***fluids*** *and various other things to keep the organs fresh”*. Instead, the vast majority of the donor relatives put forward that they were very pleased with the overall medical care but the analysis showed that they mostly could not distinguish between which treatment that aimed at recovery and which interventions that aimed at enabling organ donation.

#### Suspicion of less good medical treatment

Three relatives feared that their family member did not receive the optimal medical treatment because of the possibility of organ donation: *“Perhaps they did not give a hundred percent on this*.*”*, *“Look*, *here we have a chance*, *we might not be able to save him…”*, *“The efforts were not the same then*, *it was already a done deal apparently…”* **(now we have a chance; did not give 100 percent; a done deal)**.

### The donor operation

#### Information about the donor operation

**Unspecific information:** The information given by the staff regarding the procedures surrounding the operation was often **vague** or they **did not receive any information** according to the donor relatives: *“no they did not tell me anything*, *I mean*, *it is their thing…”*. Some relatives say that they were given information but that they **cannot recall** any of it; others **remember a few details** concerning the operation, often that the body would be restored after the operation: *“Well they told us what they would do and then restore …that they would make it look nice*.*”*

Some **did not wish additional information**. They were satisfied with the knowledge they already had concerning organ donation and put forward that they did not need any more details: “*…you have your thoughts on so many things*, *when you sit there*, *ehh…and a close family member just passed away… It was brief when she told us about it (the donor operation)*. *But I thought that it was enough what she said*. *It is one thing if you don’t know anything about organ donation*, *but I mean*, *well everyone knows*.*”*

Others clearly stated during the interviews that they **did not want to discuss** the operation. This was stated in a manner to indicate that the donor operation was something they would rather not think about: *“And how they did it*, *the donation*, *for me personally*, *it isn’t interesting*! *…No*, *I don’t want to know*, *then it becomes too much delving*, *because then you start digging into things*.*”* Among these relatives we also found individuals who expressed uneasiness when considering the operation and who also said that they had felt uncomfortable seeing the body after the donation.

**Specific information:** When the relatives refer to more specific information, it mainly **concerns the organs**; i.e. what organs can be donated and what organ is most often needed by patients waiting for an organ: *“Well*, *she told us which organs one can use*, *cornea and… I don’t know them all*, *but there are so many… But after all*, *I guess she took the organs that people… the organs that are used most often*, *what is the expression*, *longest waiting list*.*” “They try to take as much as they can*, *that is in good shape*.*”* Some had also received information about the **specialised surgeons** involved and about the comprehensive **logistic planning** that the operation requires.

#### The surgical procedure

The donor relatives’ reflections on the donor operation can be described in three distinct positions; as something **positive and/or respectful**: *“It is just like a*, *ehh*, *surgery*. *So they treat you like in any surgery… well like in any operation*. *With respect as well…”* or; something **neutral and/or with an emotional distance**: *I don’t see it as surgery you know*, *they were only going to pick them (organs) up and it is a rather complicated thing…”* and finally; as something **negative and/or connected with uneasiness**: “*How is it done*, *when they do the autopsy and all that*? *When they*, *when they take the organs and then… I don’t want them to desecrate her in any way*, *if she in some sense still would have the ability to feel or something like that*, *that I don’t know*. *But… well you know*, *one fantasises about her lying on a cold table and then they start cutting and carving*, *it mustn’t be like that…”*.

#### The state of the body after the operation

The donor relatives’ reflections on the state of the body after the donor operation can be described in the three following positions; **a positive view of the body post operation**: *“… that they were going to use the eyes sounded a bit nasty*. *But when we later took farewell of her one could not notice it at all*. *She looked so nice…”* or; **a neutral view of the body post operation:** *“No*, *she looked very tidy*, *but I don’t know if it was good or bad to see her… Yes… well… I’d rather remember her the way she looked when she was alive*. *But it is just something that you should do*, *take farewell…”* and finally; **a negative view of the body post operation**: *“That experience*, *I didn’t like that experience after the operation*. *Well*, *one collapses at once*, *when they have removed it all… She looked so collapsed*.*”*

### The donor relatives’ own decision-making process

Of the relatives who described their own willingness to donate as directly affected by their dead family member’s donation (n = 9), all but one stated that their intention had been **positively affected**; some had even changed a previously negative decision to a positive one. Having experienced the donation process made the relatives more **knowledgeable** about organ donation, and they afterwards knew that they themselves were not too old or too sick to donate. Some had also been **inspired** by knowing how many recipients had been helped thanks to their dead family member’s donation, and for some the **pride** they felt at their dead family member’s donation had inspired or reinforced their own willingness to donate. The one relative who had been **negatively affected**, decided to change her previously positive decision in protest against a chain of events during her husband’s stay at the ICU, including **treatment mistakes** made during the medical care of her husband which she believed to have contributed to his death. In addition, she also thought that her husband’s arm had been exposed in a TV-news report **(media exposure)** about organ shortage.

The decisions of most of the relatives regarding organ donation, however, were not directly influenced by their dead family member’s donation (n = 12). Instead, their minds had often been made up long before and in many cases the basis for their decision had been related to a **willingness to help others** in need, or with a belief that it was the **right thing to do**. They stated that if you are willing to receive an organ, you should also be willing to donate. Some donor relatives had knowledge of the fact that the need for organs by far exceeds the number of organs available (**organ shortage**), or they had knowledge of other facts concerning organ donation through their **employment in medical health care**. Even though those donor relatives often had made up their minds a long time ago, many stated that they now felt even more convinced and strengthened in their decision to donate. Hence, they went from an often instinctive decision, to a more conscious choice to donate.

**Fig 3:**
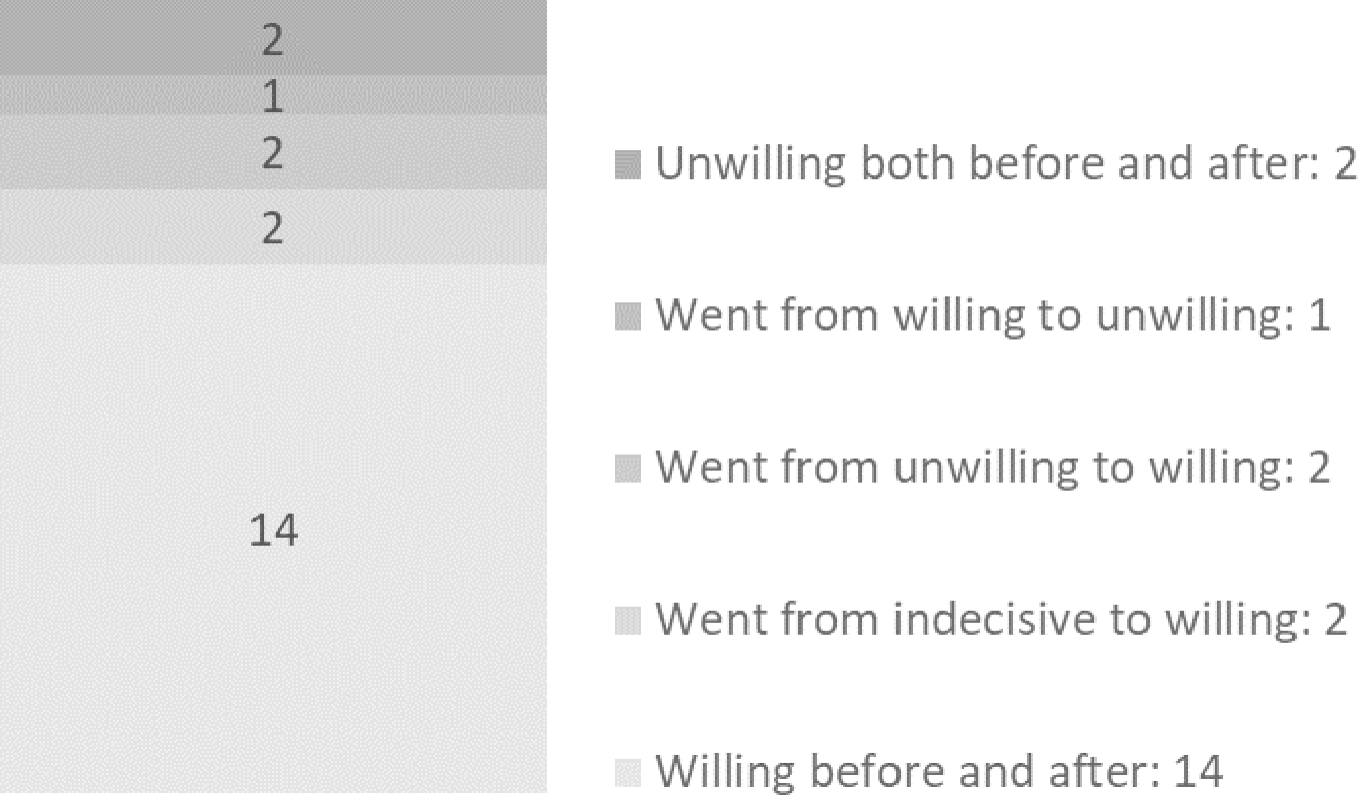
Donor relatives’ own standpoints on organ donation before and after having lost a family member who donated organs.

After having experienced the loss of a family member and the subsequent donation, the majority of the relatives remained willing to donate (n = 14, see Fig 3). However, two relatives remained unwilling to donate; one stated that he **feared that his organs would be taken before death** and the other wanted his **burial according to old traditions**, with the body intact.

## Discussion

Donor relatives represent a unique source of information about the acceptance of the medical interventions necessary to enable donation. Therefore, this study explores the relatives’ experiences of the interventions enabling organ donation. Several studies are published regarding various aspects of donor relatives’ experiences [19–22], but, to our knowledge, none have focused specifically on relatives’ experiences of the medical interventions enabling organ donation. Could these medical interventions be considered respectful, and being in the best interests of the patient, although the patient him/herself doesn’t benefit medically from it? By exploring different aspects of the relatives’ experiences and understanding, we aimed at widening our knowledge on the transition from life-sustaining treatment to organ-preserving interventions.

The trust in the medical health care in general as well as the trust in the end-of-life care, is most likely an important factor in how the donor relatives regard the necessary interventions provided during the donation process [7, 22]. In this study, the donor relatives’ trust in the medical care given during the dying and donation process has proven to be high. Most of the relatives find that the staff did everything possible in order to save their family member, and there was a gradual realisation that nothing more could be done to save their family member’s life. Only three relatives expressed doubts regarding the medical interventions given in order to enable organ donation. Nevertheless, the results indicate that there is a need for a greater transparency regarding several steps during the donation process, such as: brain death; the function of the ventilator after death; the purpose with the treatment when it is no longer intending to save the patient’s own life but to help the recipients of the organs; and the donor operation. Those areas will be discussed below.

### Donor relatives’ confusion about the actual time of death

The actual time of death of the family member was unclear to many donor relatives. One contributing factors to this confusion is most likely that the staff sometimes talked to the dead patient as if the patient was still alive, and that the staff did not use the term ‘dead’ when communicating with the donor relatives. Furthermore, some relatives were not formally informed of the time of death until they came back to the hospital after the donor operation. This underlines the importance of the concept of brain death becoming an accepted and integrated part of the ICU staff’s verbal and non-verbal communication. However, recent studies show that some ICU staff still lack trust in the concept of brain death and the clinical procedures used to determine death [23–29].

The staff most likely contributed to the relatives’ vague ideas concerning the time of death which is quite often conveyed in the interviews. This is also supported by studies conducted by others, which point out that ICU staff do feel uneasy and do lack verbal clarity when caring for donor relatives during the process of dying and donation [27, 30–32]. The donor relatives’ confusion about the time of death could potentially jeopardise the trust in the procedures necessary to make organ donation possible. However, only one relative in our study expressed explicit doubt about the validity of the way that the physician declared the family member brain dead. Yet, three relatives in our study believed that the family member was still alive at the actual donor operation and died during the operation; this could potentially cause an immense amount of discomfort for the donor relatives. Fortunately, this does not seem to be the case for the relatives in this study, as they have a high degree of confidence in the general medical care and in the procedures surrounding the donor operation. When describing death under these circumstances the staff, however, has to be unambiguous and extra thorough to ensure that the relatives truly understand that the family member is dead [19, 21]. Moreover, when a patient is dead, it is important that the staff changes its approach to the patient, without compromising dignity and respect [23–29]. A person cannot be only partially dead, he is either dead or alive, and the staff needs to make sure that they act and express themselves in accordance with the situation.

### Donor relatives’ difficulties to distinguish between the medical treatment aiming at survival and the interventions aiming at enabling organ donation

In spite of the confusion about the exact time of death, it seems that crucial to the relatives’ accepting the medical procedures surrounding the organ donation was the realisation that there was no hope for recovery, and that the death of their family member was imminent, regardless of the medical treatment given. The ventilator treatment appears to be the most obvious medical treatment provided in order to enable organ donation, and the purpose of the ventilator was quite often understood correctly, but sometimes the ventilator itself contributed to the confusion about the time of death. This as the dead patient actually looked alive even after death. In order to make it easier for the donor relatives to grasp the fact of death under these circumstances, and in order for them to be aware of and comfortable with the donation procedures, the function of the ventilator needs to be thoroughly explained.

Our analysis indicates that the confusion about the time of death also made it hard for the donor relatives to distinguish what medical treatment that was given in order to save the patient and what interventions that were given in order to maintain a high quality of organs. If this transition of care isn’t clearly defined, the staff may hesitate to be open about the intention with the provided interventions. There are also studies indicating that it is perceived as challenging to provide these interventions and that it sometimes causes discomfort for the staff [30, 33]. In our country, the fact that the patient’s intention regarding organ donation cannot be fully explored until death is confirmed, as the national donor registry cannot be accessed before death, is adding to the complexity of this situation. Furthermore, if organ donation was not a possibility, the life-sustaining treatment would be withdrawn and the fact that treatment instead needs to be prolonged to enable organ donation, is not clearly addressed in the current regulations. This is most likely also contributing to the staffs’ lack of transparency. Consequently, the interventions are seldom fully explained to the donor relatives. This may well contribute to the fact that the relatives in this study do not distinguish between what medical interventions that are aimed at organ preservation and what treatment that is provided in order to save their family member’s life. However, the vagueness of the medical interventions provided during this phase could potentially harm the trust for the care provided during the dying and donation process, as well as for the donation field in general. After death however, the regulations are clear: interventions enabling donation can then be prolonged for a maximum of 24 hours for medical and ethical reasons.

We believe that the purpose of the interventions which are beneficial not to the patient but to the recipients of the organs only, ought to be open and transparent. Hence, both ICU staff and donor relatives would most likely benefit from the physician in charge openly stating the time point for when the patient is beyond saving; when the medical interventions therefore no longer are benefitting the patient. The basis for this crucial decision should then be clearly stated and communicated to the donor relatives. A logical consequence of this routine would then be that the staff describes the function of the ventilator and discusses with the donor relatives the following two possible scenarios;

- Prolonging, for a reasonable length of time, the ventilator and organ-preserving interventions with the sole purpose of making organ donation possible, while awaiting the occurrence of brain death, and after that investigating the patient’s willingness to donate.
- Withdrawing the ventilator treatment and all futile life-sustaining care, and providing instead palliative care making the patient as comfortable as possible during the dying process.

This would then clarify the purpose of the ventilator and the fact that there is no possibility for survival, with or without ventilator. However, there is a third, very logical, option which is already practiced in some countries [2, 3]. This option is to investigate the patient’s willingness to donate at the time point when there is no hope for survival, and to proceed in accordance with the patient’s wishes:

- To prolong the organ-preserving interventions until brain death—for those willing to donate only.
- To withdraw all treatment for those not willing to donate—and instead to introduce palliative care making the patient as comfortable as possible during the dying process.

When the donor relatives are approached with the above mentioned scenarios it is important that all medical staff use the same vocabulary, and that it is clear that there is no hope for survival either way, and that the intention with the prolonged interventions, or with the withdrawal of treatment, is to respect the dying person’s willingness/unwillingness to donate organs [3, 34]. However, the fact that the donor registry in our country cannot be accessed until after death complicates the use of the two latter scenarios.

### What information is appropriate regarding the donor operation?

The information given about the donor operation varies, but in most cases the information provided by the staff seems to be limited. Some donor relatives explicitly expressed that they did not want any information about the procedure, whereas others wondered what was taking place during the many hours of surgery. We also find relatives who distanced themselves from the operation or who felt uncomfortable when thinking about it. Regarding the information provided, we believe that the responsiveness of the staff to the individual relative is of key importance. However, it seems reasonable to further discuss how the information can be drafted in a way to better describe the procedure and to help the donor relatives to come to terms with the organ procurement surgery. We believe that clear, yet individualised information reduces the occurrence of upsetting thoughts, and also helps relatives who choose to take a final farewell after the operation to prepare for this situation. According to our definition of the donation process (Fig 2), the donor operation is a natural part of the process which ought not to be neglected. The following is an example of what information donor relatives may benefit from: that the donor operation is a surgery done respectfully in an operating room, by experienced surgeons; how long the surgery may take and why the surgery often takes much time–that there are different surgeons for different organs that need to come to the hospital and remove the organs carefully in order for the organs not to be destroyed; which organs that will be removed; how the dead patient will look after the donor operation–the lengths of the incision, no ventilator, could and pale.

### What do people agree to when stating a willingness to donate organs?

In many countries there is a widespread willingness to donate organs after death [10]. However, the general public often reach their standpoint on organ donation after having been asked the question in a survey, thus they do not have full insight into the procedures surrounding an organ donation. These kinds of surveys provide important information, but what they do not address is the comprehensive medical procedures required in order for an individual to be able to donate organs. However, it seems reasonable to assume that the donor relatives in this study include their experiences of the medical treatment transitioning from being provided in order to save their family member’s life to being provided in order to make organ donation possible, in their own decisions. Some also explicitly state that their standpoint was directly affected by the loss of a family member who donated organs whereas others refer to a decision made much earlier. However, all relatives in this study have a personal experience of organ donation, and of the medical treatment provided in order to enable donation.

Though this article addresses several areas that could potentially cause donor relatives immense distress, areas that ought to be improved and clarified, such as; the information about the time of death, the ventilator and organ-preserving interventions and the donor operation. Nevertheless, most of the relatives in this study were either inspired to donate or strengthened in their willingness to donate. Hence, the medical interventions provided during the dying and donation process did not discourage the relatives from donating their own organs–but rather inspired a willingness to donate.

### Methodological considerations

There is a limitation to this study that need to be considered when interpreting the findings. The interviews were conducted in average three years after the loss which can influence the donor relatives’ ability to remember. However, we found that it sometimes was easier to remember when some time had passed, which often meant that the donor relatives had gone through the grieving process, than for those who more recently had experienced the loss. Nevertheless, the ability to remember the donation process in detail naturally varies. In order to eliminate this obstacle, the interviewer gave plenty of time for the interviews, in a safe and calm environment. By gradually guiding the donor relatives through the process at the ICU, memories often came back.

In qualitative studies, there is no need for large samples as one does not talk in terms of the sample being representative, and the findings are not to be generalized to a larger population in the same way as quantitative findings [12–16]. However, what is important is that the context and the characteristics of the sample is described, in order for the reader to decide whether the findings can be transferred to his/her own context. The variety of experiences among the donor relatives in this study is most likely to be found in other groups of donor relatives, given that the circumstances are similar. Though cultural differences, there are a lot of similarities between all countries with well-established systems for organ donation. For instance, the medical interventions necessary in order to maintain organ viability—which is the focus of this paper—is a prerequisite for organ donation regardless of regulations, country or culture.

Currently, most people have decided whether to donate their organs or not without having full insight into the procedures surrounding an organ donation. This study, however, shows that the decisions made by an informed group of the general public are positively affected by increased knowledge. Though we have encountered areas for improvement, the procedures carried out during the dying and donation process with the purpose of enabling organ donation seem to be well accepted. Nevertheless, we welcome complementary work via for example observational studies, on the communication of the interventions enabling organ donation.

## Supporting information

S1 Text Appendix, detailed statement on the ethical considerations. (DOCX)

S2 Text Interview guide in English and Swedish. (DOCX)
