# Supplementary material for: What do people agree to when stating willingness to donate? On the medical interventions enabling organ donation after death
Source: PLoS One. 2018 Aug 24;13(8):e0202544. doi: 10.1371/journal.pone.0202544 (PMC6108459; doi:10.1371/journal.pone.0202544)
Supplement: S1 Text — (DOCX) [file pone.0202544.s001.docx]

**Appendix: Detailed statement of the ethical considerations**

When planning and conducting a study like this one, with bereaved donor relatives, there are several ethical considerations that need to be made. Below we share our reasoning.

All donor relatives received detailed written information about the study before the interview. This information contained information on the overall plan and purpose of the study, the methodology, that counseling would be offered if emotional reactions would arise, information on the possibility to end the participation at any time and without explanation, and information on anonymity, etc. We consider this information to be very important, in order for the donor relatives to be aware of the implications of a possible participation and to be able to make an informed decision on whether they wanted to participate in the study.

In addition to this written information, the interviewer called all donor relatives before they decided whether they wanted to participate in the study or not, to answer any questions and describe more about the study. When a donor relative choose to participate, he/she was encouraged to choose a location where he/she could talk without being interrupted and where he/she could feel comfortable to talk about loss and sorrow. Most participants chose their own home. All through the procedure, great consideration was given to the needs of the participants.

Naturally, it is often emotionally challenging to talk about death and donation. Hence, it is of importance that the interviewer is confident and able to create a safe atmosphere during the interviews. The interviewer in this study was an experienced social worker who had extensive experience of talking about loss, and of conducting both individual and group interviews. This experience enabled her to be flexible and responsive to the donor relatives’ reactions and needs. The donor relatives were also reminded at the time of the interview, about the possibility to – at any time and without explanation – end the interview. They were also encouraged to take a break when needed. To guarantee a safe study procedure, the fellow researchers in this study was also an experienced social worker and professor with vast experience of qualitative research methodology.

At the time of the interview, after the actual research interview was ended, relatives could get answers to their questions about what happened during the time at the intensive care unit. The interviewer could also, thanks to her solid knowledge of organ donation, clarify any misunderstandings surrounding the donation process that caused the donor relatives distress. During data collection, it became clear that there were many misunderstandings and unanswered questions that needed to be addressed. When needed, the interviewer provided contact with the intensive care staff who worked during the actual donation process.

One week after the interview, all participants were contacted by the interviewer, to give them the opportunity to present their thoughts about the interview, ask questions, provide supplementary information, etc. During this phone call, the participants were offered counseling by a medical social worker if the interview caused the need for additional support. However, this was not the case. On the contrary, the donor relatives often put forward that the interview helped them process their loss.

This implies that one also needs to consider the positive aspects of participating in a study like this. To be offered the opportunity to talk through the loss and the experiences from the time at the intensive care unit is not only emotionally demanding, it can also be experienced as emotionally beneficial by donor relatives. Other research also points to the positive side effects of participating in a research interview, which corresponds well with our experience. In addition, by participating in a study like this one, relatives who need additional support can be identified. One can also argue that it is not ethically justifiable to withhold bereaved from sharing their views and having the possibility to influence the procedures during the dying- and donation process at our intensive care units. Many relatives also expressed their gratitude for being listened to and for us considering their opinions.

All through the study, the integrity of the participants was respected, and data was stored according to current legislation. In the result, no personal data or other information that could jeopardize the participants’ anonymity are included.

Finally, if the results of this study encourage greater transparency regarding the whole chain of events during the donation process, we believe this would benefit both relatives and staff. We consider this the overarching argument for the ethical benefits of this study.
