# Supplementary material for: What do people agree to when stating willingness to donate? On the medical interventions enabling organ donation after death
Source: PLoS One. 2018 Aug 24;13(8):e0202544. doi: 10.1371/journal.pone.0202544 (PMC6108459; doi:10.1371/journal.pone.0202544)
Supplement: S2 Text — (DOCX) [file pone.0202544.s002.docx]

**Interview guide**

**What do people agree to when stating willingness to donate?** On the medical treatment enabling organ donation after death

*Questions most relevant to the focus of this specific study are market with an asterisk (*). However, the whole donation process was covered in the interviews, as shown below. This made it possible to interpret the donor relatives’ answers in relation to their experiences form the whole period at the intensive care unit. This specific study focusses on the medical treatment enabling organ donation and on the donor relatives’ own stand points on organ donation.*

**Introduction to the interview guide**

The order of the questions in the interview guide may be altered or rephrased during the interview situation, in order to adhere to the language and process of the specific donor relative. Follow up questions and clarifying questions should be posed. The guide serves as a support, to make sure that all areas are covered with all donor relatives, but the interviewer should primarily be guided by responsiveness and adherence to the specific donor relative.

**Before the interview**

Instructions for the interviewer:

Ask about work, education, origin, age, family relations etc.

Describe the purpose of the interview/study.

Describe how the interview will be done, the purpose of the recorder, interview guide etc.

Inform the participant about the possibility to pause or stop the interview at any time.

Ask about questions that need to be addressed before the interview starts.

**Start of the interview**

**Circumstances:**

If you go back to the day your XXX got ill – tell me, what happened?

What happened next?

*** The medical care of the patient:**

How was the medical care?

What was:
Good?
Not good?
Could have been done better?

Something that was lacking (too little)?
Something that they shouldn’t have done (too much)?

What information did you get about the medical care provided to your XX?

What was the intention of the medical care?

Do you have any other comments on the medical care?

**Care and support for the donor family:**

How did the staff support you and care for you?

How were you treated by the staff?

What was good about the way they treated you?

What was not good?

*** Information about death**:

When did you find out that XX was dead?

What information did you receive about death?

Do you know what investigations that were made to determine death?

Did the staff mention the word ”brain dead” or ”total brain infarction”?

Had you heard about that before?

Do you know what that is – could you describe with your own words?

Was he/she dead then? When did he/she die?

What did the staff say about what would happen next? What were they going to do?

**Information about organ donation**

Were you informed about organ donation? Tell me more…

What did the person who informed you say?

Describe how the information was carried out?

Did you receive information on one or several occasions? If more than one occasion – by the same or by different persons?

Did you have possibility to ask all the questions you wanted to?

What were you wondering about?

Did you receive all the information you needed?

**The donation request:**

*How* was the question about organ donation brought up?

*When* did the staff bring the question up?

How was it for you? What were your reactions/feelings?

Did you expect the staff to bring up the question or did it come as a surprise?

Was someone (family or friend) with you when the question was brought up or were you alone?

How do you think it was for the staff to ask about organ donation?

**Decision-making process**

Tell me, how was the decision made?

What was the decision?

What led you to that decision?

Was it something specific that made you agree to/not agree to organ donation?

Was there anyone more than you involved in the decision-making process? Who?

If more than one donor relative were present:
did both of you/all of you agree on the decision at ones or did it take some time/discussion to come to an agreement? Please tell me…

Were both of you/all of you pleased with the final decision?

Did you have enough time during the decision-making process?

How much time did it take to come to a decision?

Did you ever feel pressured to say Yes to organ donation?

Had you and XX (the potential donor), ever discussed organ donation?

Did the staff ask about your personal opinion on organ donation or did they ask about XX’s (the potential donor’s) intention?

Did the staff support you during the decision- making process?

*** After the decision – donor operation:**

If *Yes* to organ donation: Did you stay at the hospital during the donor operation?

Do you know anything about the surgical procedure?

What did they do, with him/her then?

Did you receive any information about the operation?

Did you take a final farewell after the operation?

If so, at the hospital, or at the funeral, or both?

How was it for you to take this final farewell?

If *No* to organ donation: What did you do then, after the decision was made?

When and where did you take your final farewell? At the hospital, or the funeral, or both?

**Follow up:**

Did you receive an invitation from the hospital to a follow up?

Did you yourself contact the hospital afterwards?

If any of above, what did you talk about?

What information did you receive?

**Reflections/looking back:**

Tell me, now that some time has passed, what are your thought on the fact that your … donated/did not donate organs?

Was it a good decision?

Do you regret the decision/wish that you would have come to another decision?

If *Yes* to organ donation: Do you know what organs that were transplanted and how the recipients are?

If not, would you like to know?

If *No* to organ donation: is there anything that you now think of, that would have made you come to another decision?

Who was the most important provider of information during the stay at the ICU?

Who was the most important provider of care and support during the stay at the ICU?

Do you talk about what happened with family and friends?

What is your lasting impression of the time at the hospital? In summary, how was it to be at the ICU?

*** Donor relatives own decision on organ donation**

Tell me, what is your own stand point on organ donation?

When did you make up your mind?

Have you changed your mind on any occasion during life?

What are your reasons for wanting to/not wanting to donate?

**After the interview (turn of the recorder)**

How was it to be interviewed?

Is everything okay with you?

Anything that I (the interviewer) should have done differently that I ought to think about in the coming interviews?

Anything else you want to tell me?

I will get back to you via telephone, in 1-2 weeks, to find out if there are any new questions, thoughts or reactions. You can also call me if you think of anything.

Thank you for your time and valuable contribution to an important field of research!

**Interview guide in Swedish:**

**Innan intervjun påbörjas**

Fråga lite om arbete, utbildning, härkomst, ålder, familjeförhållanden mm…

Beskriv syftet med intervjun

Beskriv hur intervjun kommer att gå till, bandspelare, guide mm

Förklara att intervjupersonen kan pausa eller avbryta intervjun när som helst

Har intervjupersonen några frågor innan intervjun påbörjas?

**Intervjuguide anhörigstudie**

Om vi går tillbaka till den dagen din…insjuknade…

**Omständigheter:**

…berätta, vad var det som hände?

**Vård:**

Hur var vården av …har du några synpunkter?

Vad var bra? Kunde något ha gjorts bättre?

Var det något som fattades beträffande vården, när ni var på sjukhuset?

**Bemötande / Omhändertagande av familjen:**

Hur tog dom hand om er då? / Vad fick ni för stöd?

Berätta, hur blev du / ni bemötta av personalen på sjukhuset?

Vad var bra i personalens omhändertagande av dig / er?

Vad var inte bra i personalens omhändertagande av dig / er?

**Information om dödsdiagnosen:**

När ni fick veta att … var död, vad sa den som informerade er då?

Vilka undersökningar gjordes?

Vad sa de skulle hända sen, vad skulle de göra?

Nämnde de ordet hjärndöd eller total hjärninfarkt?

Hade du hört talas om det tidigare?

Vet du vad det är då?

Var…död då? / När dog hon/han?

**Information om möjligheten till donation:**

Vad sa den som informerade er om organdonation?

Hur gick informationen till?

Fick ni information vid upprepade tillfällen? I så fall, av samma eller olika personer?

Hade ni möjlighet att ställa de frågor ni ville?

Fick ni tillräckligt med information?

**Donationsfrågan:**

Hur togs frågan / info om tidigare beslut / om organdonation upp?

När kom frågan om organdonation upp?

Hur var det? Hur kände du då?

Väntade du dig frågan eller kom den oförberett?

Hade du någon hos dig när frågan togs upp?

Har du någon uppfattning om hur det var för den som ställde frågan / informerade er om tidigare beslut?

Kändes det som den personen förstod er?

**Beslutsprocessen / Donationsbeslutet:**

Berätta, hur fattades ”beslutet”?

Vad kom ni fram till?

Vad var det som gjorde att du/ni sa ja/nej?

Var det något speciellt som var avgörande för att ni fattade det beslut ni gjorde?

Var det fler som var involverade i beslutet / samtalet / infon, i så fall, vilka?

Om ni var flera som var involverade i beslutet, var ni genast eniga kring beslutet, eller enades ni efter diskussion?

Om beslutet fattades efter en diskussion, kom ni då fram till ett beslut som ni alla var nöjda med?

Fick du den tid du ville för att ta ställning?

Hur lång tid?

Kände du dig någon gång pressad att säga ja?

Hade du och din anhörige tidigare diskuterat organdonation?

Uppfattade du att du skulle uttolka den avlidnes vilja eller att det var din uppfattning man frågade efter?

**Efter beslutet:**

Om ja till donation: Stannade ni kvar på sjh under operationen?

Vet du vad de gjorde med honom/henne då?

”Såg du”, din anhörige på sjukhuset efter operationen?

När tog ni farväl? På sjukhuset eller innan begravningen, eller både och?

Om nej till donation: Vad gjorde ni sen, efter beslutet?

När tog ni farväl? På sjukhuset eller innan begravningen, eller både och?

Fick ni återbesökstid?

Har ni själva tagit kontakt?

Vad pratade ni i så fall om då?

**Eftertanke:**

Berätta, hur ser du på / ditt beslut / att det blev / att det inte blev donation / så här i efterhand?

Var det ett bra beslut?

Ångrar du ditt att det blev / att det inte blev / en donation? / Önskar du att du fattade ett annat beslut än det du gjorde?

Om nej till donation, finns det något som du nu i efterhand kommer på som hade fått dig att fatta ett annat ”beslut”?

Pratar ni om beslutet och det som hände, med era vänner?

Vem var den viktigaste ”informatören” på sjukhuset?

Vem var den viktigaste stödpersonen på sjukhuset?

Hur ställer du dig själv till att donera organ?

Vad är ditt bestående intryck av tiden på IVA? Ja, hur var det?

Vet du vilka organ man har tagit tillvara och hur det går för patienterna idag?

Om nej, vill du veta?

Jag har inga fler frågor, har du något mer du vill ta upp innan vi avslutar intervjun?

**Efter intervjun:**

Hur kändes det att bli intervjuad?

Är allt okej?

Hur upplevde du intervjun?

Något jag skulle ha gjort annorlunda?

Något du vill tillägga?

Jag kommer att återkomma till dig om 1-2 veckor för att höra efter om du har några frågor, tankar eller reaktioner. Du kan också ringa mig om du undrar något eller kommer på något i efterhand.

Tack för din tid och för att du bidrar till ett angeläget forskningsområde!
